# Appraisal of clinical practice guidelines on community-acquired pneumonia in children with AGREE II instrument

**DOI:** 10.1186/s12887-016-0651-5

**Published:** 2016-08-02

**Authors:** Zhenwei Xie, Xiaoling Wang, Lin Sun, Jun Liu, Yan Guo, Baoping Xu, Libo Zhao, Adong Shen

**Affiliations:** 1 Department of Pharmacy, Beijing Children’s Hospital, Capital Medical University, Beijing, 100045 China; 2 School of Pharmaceutical Science, Peking University, Beijing, 100191 China; 3 Key Laboratory of Pediatric Respiratory Infection Diseases, Beijing Pediatric Research Institute, Beijing Children’s Hospital, Capital Medical University, Beijing, 100045 China; 4 Department of Respiratory, Beijing Children’s Hospital, Capital Medical University, Beijing, 100045 China

**Keywords:** Clinical practice guidelines, Community-acquired pneumonia, Children, Quality assessment, AGREE II

## Abstract

**Background:**

Community-acquired pneumonia (CAP) remains a major cause of morbidity and mortality worldwide among children. The growing number of guidelines have been accompanied with a growing concern about variance and conflicts among guideline recommendations. There is a need to critically appraise clinical practice guidelines (CPGs) in order to ensure safe and effective practices.

**Methods:**

A literature search was systematically conducted in English and Chinese major academic databases (from January 2000 to March 2015). CPGs related to CAP in children were evaluated by four independent assessors, according to AGREE II instruments. Standardized domain scores were calculated for each guideline. Inter-rater reliability was assessed by intraclass correlation coefficient. The software used for analysis was SPSS 17.0.

**Results:**

A total of 10 guidelines met the inclusion criteria and were appraised. Scope and purpose (69.03 %) and clarity of presentation (83.33 %) achieved relative high scores, while the scores of the other four domains were low: stakeholder involvement (42.78 %), rigour of development (44.95 %), applicability (37.60 %), and editorial independence (23.74 %). 3 guidelines were strongly recommended as a result of the overall scores were greater than 60 %.

**Conclusion:**

The qualities of CPGs for CAP in children were generally acceptable with several flaws. Stakeholder involvement, rigour of development, applicability and editorial independence should be considered and well described in the future development of CPGs.

## Background

Community-acquired pneumonia (CAP) is defined as an acute infection of lower respiratory tract occurring outside hospital. It remains a major cause of morbidity and mortality among children in developing and developed countries. In developing countries, CAP has an incidence of 0.29 episodes per child-year and a mortality rate of 1.3–2.6 % [1, 2]. The incidence of CAP is lower in developed countries, with an incidence of about 0.05 episodes per child-year, however, it is still one of the leading causes of hospitalization [3, 4]. In certain conditions, it would occur repeatedly and cause harm to children without proper and complete treatments. Meantime, it will bring heavy burden for national public health care systems.

Guidelines for the management of CAP in children have been increasingly produced and disseminated in recent years. Generally, globalization and uniformity between centers worldwide in patient management has become a trend. However, acceptance of evidence-based medicine (EBM) varies around the world. The growing number of guidelines has been accompanied with a growing concern about variance and conflicts among guideline recommendations. Therefore, there can be huge differences between countries or regions in respect to guideline qualities. Hence, it is urgent to identify existing high quality guidelines and make guideline developers follow methodological standards while developing guidelines.

The appraisal of guidelines for research and evaluation II (AGREE II), consisting of 6 domains covering 23 key items, is initially developed from AGREE and updated in 2010. The purpose of this instrument is to evaluate the strengths and limitations of guidelines; to provide methodology for development of guidelines; and to present guidance for reporting recommendations. It is a widely-used instrument and remains the current golden standard for guideline appraisal [5, 6].

The quality of CAP guidelines has not been systematically evaluated till now. Thus, the aim of this study was to access the available guidelines for the treatment of children with CAP and to determine the methodological quality and reliability of guidelines.

## Methods

### Literature search

A literature search was systematically conducted in English and Chinese major academic databases (from January 2000 to March 2015). English databases were MEDLINE, EMBASE, Guidelines International Network (GIN), National Guideline Clearinghouse (NGC), Chinese databases were Chinese Biomedical Literature Database (CBM), China National Knowledge Internet (CNKI), VIP Database and Wanfang Database. Key words were “community-acquired”, “pneumonia”, “children”, “pediatric”, “guideline”, “guidance”, “recommendation”, “management”, and “consensus”.

### Inclusion and exclusion criteria

Guidelines published in Chinese or English, which addressed diagnoses and treatments of CAP for children under 18 years of age were included. Guidelines that are systematic reviews, editorials, short summaries, and other literature explaining or evaluating guidelines were excluded. If guidelines have been fully updated, the latest version were evaluated and the old versions were excluded.

### Data extraction

Two of the investigators (Z.W.X and L.B.Z) searched the database, selected guidelines independently and determined if they warranted inclusion in this study and then extracted general characteristics of the guideline. Disagreements were resolved by consulting a third expert opinion (A.D.S). The following information was extracted, such as title of guidelines, country or region, publication date, affiliation or organization, developed method, quality of evidence and filled in an established standard table on Microsoft Excel 2013.

### Appraisal guidelines

The AGREE II instrument was used to assess the methodological quality of included guidelines. It contains 23 key items of 6 domains, such as scope and purpose, stakeholder involvement, rigour of development, clarity of presentation, applicability, editorial independence, and overall guideline assessment. Each item was scored on a scale of 1–7, with 1 being strongly disagree and 7 being strongly agree. Each domain was calculated by summing up all the scores of the individual items in a domain and then standardizing as follows: (obtained score - minimal possible score) / (maximal possible score - minimal possible score).

Quality assessment were performed independently by four trained reviewers (Z.W. X, L.S, J.L, and Y.G). They conducted an independent review of the quality of each eligible guideline. Disagreement between reviewers was resolved through consensus or by consulting an independent expert adjudicator (L.B.Z). Final average appraisal scores and standard deviations for each domain and scaled domain percentages were calculated.

Intraclass correlation coefficient (ICC) was used as a measure of agreement between reviewers. The ICC values was carried out by SPSS v.17.0 and applied to each guidelines.

Finally, the overall guideline assessment was evaluated. A guideline would be “recommended” if overall scores were greater than 60 %, “recommended with modifications” at scores between 30 % and 60 %, and “not recommended” at scores less than 30 %.

### Statistical analysis

Standardized domain scores were calculated according to methods described in the AGREE II user manual. A descriptive statistical analysis was used to summarize the data. Subgroup comparison between evidence-based guidelines and non evidence-based guidelines were performed by t-test. All tests were two-sided, and *P* values < 0.05 were considered statistically significant. All statistics were calculated using SPSS v.17.0.

## Results

**Fig. 1:**
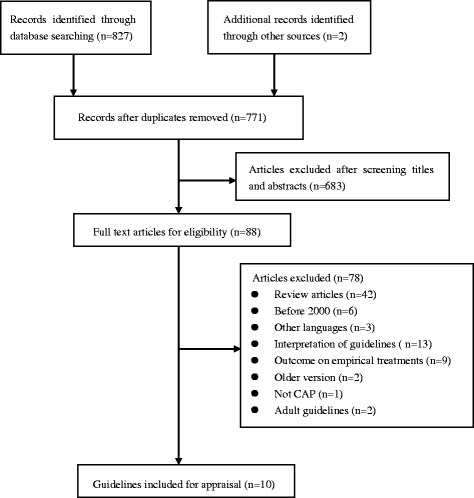
Flow chart of searching and selecting guidelines

A total of 827 records were identified through comprehensive database search and 22 additional records more were identified through Google search engine. 56 were exclude due to duplication, and the remaining 771 were screened by titles and abstracts. 88 were screened by full text articles for eligibility. 42 review articles, 6 guidelines before 2000, 13 interpretation of guidelines, 9 outcome on empirical treatments, 2 old version, 1 involving other disease guideline, 2 adult guidelines, 2 old version (Canadian 97, and Thorax 2009) and 3 other foreign languages (Brazilian, Spanish and Italian guidelines) were excluded. Finally, 10 guidelines were identified and involved in the analysis [7–17]. The details of review process was summarized in Fig. 1. The characteristics of each included guideline were presented in Table 1.

**Table 1:** Characteristics of 10 included guidelines

Clinical practice guideline	Country or region	Language of guidelines	Publication date	Affiliation or organization	Developed method	Quality of evidence
[7]	United States	English	2011	Pediatric Infectious Diseases Society and the Infectious Diseases Society of America	Evidenced-based	GRADE
[8]	United Kingdom	English	2011	British Thoracic Society Community Acquired Pneumonia in Children Guideline Group	Evidenced-based	Self designed grading system
[10]	South African	English	2005	Working Groups of the Paediatric Assembly of the South African Thoracic Society	Literature review	Not mentioned
[13, 14]	China	Chinese	2013	Subspecialty Group of Respiratory Diseases, The Society of Pediatrics, Chinese Medical Association	Literature review	Self designed grading system
[16]	Malaysia	English	2004	Ministry of Health Malaysia Academy of Medicine Maylaysia	Evidenced-based	SIGN
[12]	Taiwan	English	2007	Taiwan Pediatric Working Group for “Guideline on the management of CAP in children”	Literature review	Not mentioned
[9]	Japan	English	2007	Japanese Society of Pediatric Pulmonology and the Japanese Society for Pediatric Infectious Diseases	Literature review	Not mentioned
[15]	WHO	English	2013	Word Health Organization	Literature review	Not mentioned
[11]	Canada	English	2011	Canadian Paediatric Society, Infectious Diseases and Immunization Committee	Literature review	Not mentioned
[17]	United States	English	2006	Cincinnati children’s hospital medical center	Evidenced-based	Self designed grading system

### Scope and purpose

This domain addressed the overall objectives, health issues and target population. The score of this domain was relatively high (mean 69.03 %, range: 23.61–95.83 %), indicating most guidelines met the criteria of this domain.

### Stakeholder involvement

This domain assess whether the relevant professional group is represented, the patients’ views and preference has been sought, and the definition of target users of the guideline are clearly defined. The score of this domain was 42.78 % (range: 9.70–76.39 %). A few guidelines [7, 8, 10, 13, 16, 17] could describe the relevant professional group, however, few guidelines seek the preference of target populations.

### Rigour of development

This domain is the core of guideline methodology and mainly focus on the method of evidence search, grading, summarizing and the formulation of the recommendation. The score of scope was 44.95 % (range: 17.19–79.69 %).

### Clarify of presentation

This domain evaluates the presentation and the format. The score of this domain was 83.33 % (range: 69.44–94.44 %). Most guidelines recommendations were specific. Different options for management of the condition were clearly presented, and key recommendation was easily identifiable.

### Applicability

This domain discusses the organization barriers, cost implications and monitoring criteria. The assessment result was 37.50 % (range: 17.71–61.46 %). Most guidelines failed to consider it during the guideline development.

### Editorial independence

This domain focuses on the funding issues and competing interests of all contributing members. The score of this domain was 23.74 % (range: 0–87.50 %). Both evidence-based guidelines and non evidence-based guidelines achieved low scores in this domain.

### Agreement among reviewers

ICC for each guideline is listed in Table 2. Eight of 10 ICC values were (>75 %) excellent. No ICC values were less than 45 %. The reliability of this reviewers were acceptable.

**Table 2:** Standardized scores of each domain by AGREE II of guidelines

Guideline	Scope and purpose (%)	Stakeholder involvement (%)	Rigor of development (%)	Clarity of presentation (%)	Applicability (%)	Editorial independence (%)	ICC	Overall assessment
[7]	95.83	73.61	79.17	94.44	54.17	87.50	0.82	Strongly recommended
[8]	93.06	37.50	79.69	93.06	61.46	58.33	0.73	Strongly recommended
[10]	81.95	34.72	32.29	76.39	35.41	33.33	0.88	Recommended with modifications
[13, 14]	80.56	45.83	30.21	80.56	33.33	0	0.88	Recommended with modifications
[16]	79.17	76.39	60.94	86.11	33.33	0	0.84	Recommended with modifications
[12]	23.61	11.11	19.27	86.11	17.71	0	0.91	Not recommended
[9]	61.11	9.70	23.44	83.33	25.00	0	0.91	Not recommended
[15]	52.78	48.61	32.81	73.61	35.42	29.1	0.69	Recommended with modifications
[11]	30.56	15.28	17.19	69.44	31.25	0	0.86	Not recommended
[17]	91.67	75.00	74.48	90.28	47.92	29.17	0.82	Strongly recommended
Mean (range)	69.03 (23.61–95.83)	42.78 (9.70–76.39)	44.95 (17.19–79.69)	83.33 (69.44–94.44)	37.50 (17.71–61.46)	23.74 (0–87.50)	/	/

### Overall quality assessment of guideline

**Fig. 2:**
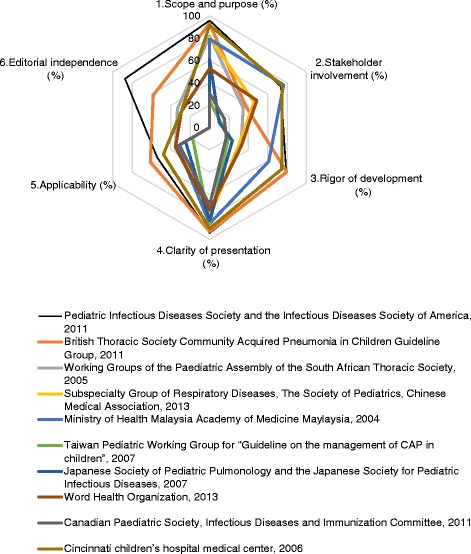
AGREE II domain scores are plotted for each guideline for comparison

3 CPGs [7, 8, 17] were strongly recommended as results of the overall scores greater than 60 %. 4 CPGs [10, 13, 15, 16] were “recommended with modifications”, whose scores between 30 % and 60 %. 3 CPGs [9, 11, 12] were “not recommended” due to all items scores being less than 30 %. Finally, IDSA guideline [7] achieved a relative high score among the 10 guidelines. This provides a reference of methodology for developing other CAP guidelines. The details of the appraisal results are presented in Table 2. AGREE II domain scores are plotted for each guideline for comparison are showed in Fig. 2.

Of the guidelines involved, 4 were evidence-based guidelines which processed details of evidence scale, on the other hand, the remaining 6 were more consensuses than evidence-based guidelines. Compared with non evidence-based guidelines, evidence-based guidelines obtained significant higher scores in nearly all domains except “editorial independence”. Table 3 shows a subgroup analysis of the involved guidelines.

**Table 3:** Comparison of mean quality scores among evidence-based guidelines and non evidence-based guidelines

Subgroup	Domain (Mean ± SD)					
	Scope and purpose (%)	Stakeholder involvement (%)	Rigor of development (%)	Clarity of presentation (%)	Applicability (%)	Editorial independence %
Evidence-based guideline	89.93 ± 7.38	65.63 ± 18.78	73.57 ± 8.74	90.97 ± 3.67	49.22 ± 11.95	43.75 ± 37.65
Non Evidence-based guideline	55.10 ± 24.51*	27.54 ± 17.71*	25.87 ± 6.83*	78.24 ± 6.25*	29.69 ± 7.02*	10.41 ± 16.17

Data are presented as mean ± standard deviation. *P < 0.05

## Discussion

This study discussed a qualitative evaluation of the CPGs on CAP in children with the AGREE II guideline assessment. Eventually, 10 guidelines were included. In this study, we demonstrated that the scores of CAP guidelines were higher than an average international quality level [18], especially “Scope and purpose” and “Clarity of presentation”. Compared to the guidelines published from 1980 to 2007, current guidelines could specifically describe the purpose of the guidelines and also clearly present the recommendation. However, in some domains the scores were much lower when compared with contemporary guidelines in respect to pediatric infectious diseases [19], such as “rigour of development” and “editorial independence”. The average score of the evidence-based guidelines on pediatric infectious diseases respectively was 76.04 % and 81.25 %. Hence, there is still potential improvement with respect to qualities of pediatric CAP guidelines.

It was concluded from our results that CPGs tackling CAP on children account for a small amount compared with adults. A great number of countries or associations ignored to develop guidelines which are targeted to this particular group. Pediatric patients are tend to be more vulnerable in nature, therefore, it is essential to ensure that guidelines are developed under strict criteria. On the other hand, a great number of organizations failed to translate the CAP guidelines into English and hindered the efficiency of health science communication. Efforts are needed to translate guideline into a universal language and to assist health professions in better mastering strictly developed guidelines and to rectify discrepancies and promote evidence-based practices to enhance patient outcomes.

In our study, it should be noted that “scope and purpose” and “clarity of presentation” achieved a relative high scores among the six domains, which indicated that most guidelines fully satisfied this criteria. Most guidelines could fully describe the overall objective and their specific clinical issues. For “stakeholder involvement”, relevant professionals in the development process were involved in most guidelines and target population was declared. However, the guideline developers did not seek the preference of target populations resulting in a decrease in score of this domain.

“Rigour of development” was considered to be one of the most crucial domain for the assessment of guideline development. In our study, this domain varied in guidelines, for evidence based guideline, it obtained higher scores, while consensus not. A strong recommendation should not solely be based on expert opinion. Guideline developers should adhere to a standardized evidence-based evaluation system. In this study, only 4 [7, 8, 16, 17] guidelines clearly described the criteria for selecting the evidence, as well as strengths and limitations of the evidence. The quality may be improved by involving methodologists in the guideline development. It is also expected that CPGs should grade the quality of evidence on an existing well recognized grading system such as GRADE (Grading of Recommendations Assessment, Development and Evaluation). GRADE grading system was first introduced in 2004 for access the quality of evidence and strength of recommendation. Gradually, it was recognized by more than 30 organizations including WHO and Cochrane Collaboration. Although the 10 guidelines included in our study were published after 2005, only 1 CPG [7] use the GRADE system. 1 CPG [16] use SIGN (The Scottish Intercollegiate Guidelines Network) and 3 CPGs [8, 13, 17] guidelines used their own developing system. We suggest further guidelines should use a comparable uniform grading system to evaluate the quality of evidence and strength of recommendations. In this domain, a procedure of updating the guidelines was neglected in most guidelines. Only 2 CPGs [16, 17] referred the updating procedures. It is recommended that guidelines be updated at 3-year intervals, as new evidence may result in substantial changes to the recommendations [20].

Our study found that “applicability” scored low among the six domains. A great number of guidelines lack of describing facilitators and barriers to its application, advice and/or tools on how the recommendations can be put into practice. Guideline would rate high if the potential resource implications of applying the recommendations had been considered, such as a summary document, a quick reference guide, and educational tool. In order to ensure the feasibility of clinical practice, it is also suggested that a pilot test for the applicability of new guidelines should be performed before its publication. These items should be taken into consideration when the following guidelines are developing.

Editorial independence was also neglected in a great deal of guidelines. Only 3 [7, 10, 17] guidelines reported detailed information on potential conflicts of interest. Conflicts of interest are the most common source of bias in guideline development [21]. There should be an explicit statement that all group members have declared whether they have any competing interests. It may be suggested by making it mandatory for guideline developers to list the potential competing interests in the documents.

A strength of our study is that we used AGREE II instrument to assess the methodological quality of guidelines related to CAP in children, which has no related appraisal research on this particular group. Furthermore, our team consisted of pediatric clinical experts and pharmacist with extensive experience in clinical guidelines appraisal.

There are limitations within this study that should be noted. Firstly, the guidelines involved are confined to English and Chinese. Secondly, the literature search only identified published guidelines in pediatric pneumonia. It is possible that some guidelines may have been missed by the search, especially those that combined pediatric and adult recommendations. Thirdly, the AGREE II instrument is a tool that assesses the methodological rigour and transparency in which a guideline is developed [22, 23]. It informs what information and how information ought to be reported in guideline. However, there is no threshold for discrimination from high quality to low quality CPGs. Thus, the guidelines quality would be left to the appraisers to identify and the scores of an AGREE II evaluation have to be interpreted with caution and confined to a particular situation.

## Conclusion

Overall, this study appraised quality scores of the CPGs on CAP in children. The appraisal results showed acceptable. The developers are suggested to pay more attention to rigour of development, applicability, and editorial independence during the development process. The AGREE II instrument should be adopted by guideline developers to improve the applicability of guidelines.

## Abbreviations

AGREE II, the appraisal of guidelines for research and evaluation II; CAP, community-acquired pneumonia; CBM, Chinese databases were Chinese Biomedical Literature Database; CNKI, China National Knowledge Internet; CPGs, clinical practice guidelines; EBM, evidence-based medicine; GIN, Guidelines International Network; GRADE, Grading of Recommendations Assessment, Development and Evaluation; ICC, intraclass correlation coefficient; IDSA, Infectious Diseases Society of America; NGC, National Guideline Clearinghouse; SIGN, The Scottish Intercollegiate Guidelines Network
